# HIV test uptake and related factors amongst heterosexual drug users in Shandong province, China

**DOI:** 10.1371/journal.pone.0204489

**Published:** 2018-10-18

**Authors:** Zhenxia Jiang, Cuizhen Xiu, Jun Yang, Xijiang Zhang, Minghua Liu, Xinlong Chen, Dianchang Liu

**Affiliations:** 1 Department of HIV/STI Prevention and Control, Qingdao Municipal Center for Disease Control and Prevention, Qingdao, Shandong, China; 2 Department of STI and Leprosy Prevention and Control, Jining Hospital for Skin Disease Control and Prevention, Jining, Shandong, China; 3 Department of STI and Leprosy Prevention and Control, Shandong Provincial Institute of Dermatology and Venereology, Jinan, Shandong, China; Pontificia Universidade Catolica do Rio Grande do Sul, BRAZIL

## Abstract

**Background:**

In China, the mode of HIV transmission and the types and routes of drug use have changed in the past decade. HIV testing amongst drug users needs further improvement.

**Methods:**

Interviewer-administered questionnaires were used for data collection amongst 600 heterosexual drug users from the community and a municipal detention centre, where criminal suspects are supervised. Descriptive statistics, univariate analysis and multilevel logistic regression analysis were used to identify the factors associated with HIV testing amongst heterosexual drug users. All participants were screened HIV and sexually transmitted infections.

**Results:**

Amongst 600 participants, 554 (92.3%) were methamphetamine users, and 155 (25.8%) underwent HIV testing in the past year. Multivariate analysis results revealed that drug users who were single (adjusted odds ratio(AOR) = 1.923, 95% confidence interval(CI) = 1.189–3.109), had less knowledge of HIV (AOR = 1.706, 95% CI = 1.074–2.711), used only one kind of drug (AOR = 2.649, 95% CI = 1.155–6.077), used drug via a non-injection route (AOR = 2.121, 95% CI = 1.103–4.078), did not receive free condoms (AOR = 2.307, 95% CI = 1.129–4.715) and who did not receive free publicity material from health workers in the past year (AOR = 2.828, 95% CI = 1.757–4.552) were less likely to undergo HIV testing. A total of 594 participants underwent HIV and syphilis screening in this survey. Amongst these participants, 2 (0.3%) were HIV antibody positive, and 88 (14.8%) showed positive results in both non-treponemal test (rapid plasma regain test) and treponemal test (*Treponema pallidum* particle agglutination test) for the first time.

**Conclusions:**

The rate of HIV test uptake amongst heterosexual drug users in China remains low. Thus, more specific interventions are urgently needed to improve the utilisation of HIV testing amongst heterosexual drug users, particularly amongst non-injection drug users in China.

## Introduction

Amongst drug users, injection drug use is strongly associated with HIV infection[1].Injection drug users used to be one of the main subpopulations affected by HIV in China [2].The prevalence of HIV has reached as high as 12.55% amongst injection drug users in China, with 3.73–4.29-fold higher odds ratio (OR) than other drug users [3, 4].However, in the past decade, sexual transmission has become the primary mode of HIV transmission in China [5].The percentage of HIV-infected people who inject drugs has become stable and slightly declined in China, decreasing from 6.33% in 2013 to 6.00% in 2014 [5].Amongst new cases diagnosed each year, the percentage of cases transmitted by injection drug use decreased from 34.1% in 2006 to 6.0% in 2014, whereas the percentage of sexually transmitted cases increased from 33.1% in 2006 to 92.2% in 2014 [5].

In recent years, the importance of non-injection drug use in HIV transmission has increased considerably because of its association with increased sexual transmission and the increased number of non-injection drug users. In the past decade, amphetamine-type stimulants, such as methamphetamine (MA) and methylamphetamine, have replaced traditional injectable drugs and become the most well-known hazardous drugs globally [6]. In China, the proportion of registered synthetic drug users (used to be arrested and registered by the police) increased from 9.5% in 2004 to 60.5% in 2016[7, 8]. Studies have shown that MA use is strongly associated with all kinds of high-risk sexual behaviour [9, 10].

HIV voluntary counselling and testing (VCT) is a highly cost-effective intervention for HIV transmission and risk reduction, as it can increase knowledge on HIV/AIDS, reduce unsafe sexual and drug use behaviour and prevent other sexually transmitted infections (STIs) [11–16]. VCT is an effective strategy that decreases HIV-related risk and increases protective sexual behaviour amongst HIV-infected individuals in low-income and middle-income countries [17]. It is estimated that 49% of new HIV cases are transmitted by people who are unaware of their serostatus [18]. Although China has continued to implement the major response measures of ‘Five Expands and Six Strengthens’ (‘Five Expands’ means to expand information, education and communication activities; surveillance and testing; prevention of mother to child transmission; comprehensive interventions; and coverage of antiretroviral therapy.‘Six Strengthens’ means to strengthen blood safety management, health insurance, care and support, rights protections, organisational leadership and strengthening of response teams) and has achieved marked progress with regard to expanding testing and counselling services in recent years, (e.g. an estimated 130 million person times of HIV antibody testing were conducted in 2014, an increase of 70 million person times than that in 2010), further improvement is still necessary [5]. In China, the prevalence of HIV amongst the general population was estimated at 0.06%, and 32.1% of the infections were not detected by the end of 2015[19]. By the end of June 2016, 627,030 living individuals were reported to be infected with HIV/AIDS [20]. A survey in Shanghai, China reported that only 24.4% of injection drug users agreed to be tested for HIV [21]. To date, estimates of HIV infection amongst non-injection drug users are generally unknown. Changes in the types of drug use and mode of HIV transmission call for new approaches to control HIV infection amongst drug users. Given that comprehensive HIV testing and prevention services in China are continually being scaled up but remain inaccessible to many drug users, improving the understanding on the barriers to these prevention services is urgently needed. The current study was conducted to investigate the utilisation of HIV testing and related factors that influence heterosexual drug users, particularly non-injection drug users, in Qingdao, China to undergo HIV testing.

## Materials and method

### Setting and participants

The study was approved by the Ethics Review Committee of Shandong Provincial Institute of Dermatology and Venerology. The approval number SPIDV-2016-012. The study was conducted in December 2016, in Qingdao, Shandong Province, China. Qingdao is one of the economically developed coastal cities in China. In the past decade, it has become known as a distribution centre for drugs from South Korea, Japan and Russia, where drug trafficking and abuse have become much common.

The participants were recruited from a municipal detention centre where criminal suspects are supervised, and from the community. The inclusion criteria included (1) individuals detained due to drug use in the detention centre or used drug once a month at least in the past year in the community (2) and those who self-identified heterosexuals. The exclusion included the following: (1) individuals who refused to respond (2) those that self-identified as homosexuals.To avoid confounding for homosexual factors, men who had sex with men (MSM) were excluded in this study.

A key informant, doctor W, played an important role in data collection. He was a dermatologist in the community hospital and also one of our researchers. The community hospital engaged in providing health service for all detainees in the detention centre. Doctor W was responsible for infectious disease screening and treatment in the detention centre. With the permission of the detention centre staff and detainees, we interviewed each newly registered drug user. The ‘snowball’ approach was applied in the enrolment of the participants from the community. Firstly, we recruited 11 key informants as seeds from the local STI clinic or from the community; secondly, we encouraged the seeds to introduce more partners to visit the clinic; thirdly, we visited the entertainment venues or dwelling where drug users gathered by outreach.

An agreement of participation was signed by all participants before the interview. Before the interview with the detainees, we introduced ourselves at first and assured them that the survey had no relation to their crimes and punishment. They had right to refuse or quit at any time. All participants were provided with adequate information about the study, including confidentiality and use of the data, so that they could make informed and voluntary decisions for participation. Informed consents were obtained from the parent of participants under 18 years old in the detention centre. In addition, the participants were anonymous to ensure the confidentiality of the information they would provide.

All participants recruited were requested to undergo HIV antibody screening via an enzyme-linked immunosorbent assay test (InTec Products, Xiamen, China) and syphilis screening via a rapid plasma regain (RPR) test (Shanghai Rong Sheng Biostix Inc., Shanghai, China) at the community centre for disease prevention and control.

It is compulsory for all detainees to take these tests in China. Participants who tested positive were further confirmed by Western blot test (HIV Blot 2.2 WBTM, Genelabs Diagnostics, Singapore) and *Ttreponema pallidum* particle agglutination (TPPA) test (Shanghai Rong Sheng Biostix Inc., Shanghai, China). All participants were also given physical examination for STIs. Suspected cases of STIs were referred to an STI clinic for free treatment.

### Data collection

Interviewer-administered questionnaires were used to obtain data. The questionnaire was developed based on our preliminary study amongst drug user population [22]. The format and some items in the questionnaire referred to the preliminary study. A pilot study was conducted both in the community and the municipal detention centre prior to formal investigation, and some items were adjusted. In this survey, the following characteristics of the participants were examined: demographics, perception of HIV, MA use behaviour and high-risk sexual behaviour. Each topic area was covered by several variables, and each variable was given in the form of a question and tested in the pilot investigation. The variable ‘perception of HIV’ was measured by using eight questions, and percentage of correct answers was calculated.

### Statistical analysis

The data were checked for double entries. The proportion of drug users who were tested for HIV in the past year was calculated. Descriptive statistics, univariate analysis and multilevel logistic regression analysis (Backward: LR) were performed to identify the factors associated with HIV test amongst heterosexual drug users. OR and 95% confidence intervals (CIs) were reported. SPSS version 17.0 (SPSS, Inc., Chicago, IL, USA) was used to analyse the statistical data. P<0.05 was considered statistically significant. ORs with 95% CI were reported.

## Results

### Demographic information of the participants

A total of 386 individuals from the municipal detention centre and 230 individuals from the community were interviewed. However, five individuals from the municipal detention centre and nine from the community refused to respond. Two MSM from the municipal detention centre were excluded from the analysis. Thus, a total of 600 heterosexual drug users were recruited in this study, including 379 (63.2%) participants recruited from the municipal detention centre and 221 (36.8%) from the community. Amongst the participants recruited from the community, 194 were referred to the STI clinic by a ‘snowballing’ approach.

The average age of the participants was 33.3 ± 9.1 (16–61) years. Of these participants, 488 (81.3%) were males, and 112 (18.7%) were females; 435 (72.5%) were married, 116 (19.3%) were single, and 49 (8.2%) were divorced or widowed; 411 (68.5%) were local permanent residents, and 189 (31.5%) were from other cities in Shandong Province or from other provinces; 574 (95.7%) were ethnic Han, and 26 (4.3%) were from other ethnicities; 289 (48.2%) had junior school education, 175 (29.2%) high school or technical secondary school education, 74 (12.3%) primary school education, 51 (8.5%) junior college (or above) education and 11 (1.8%) were illiterate; and 340 (56.7%) were employed, and 260 (43.3%) unemployed.

### Perception of HIV amongst heterosexual drug users

Eight questions were asked to examine perception of HIV amongst the participants (Table 1). A total of 398 (67.3%) participants answered six or more questions correctly.

**Table 1:** Perception of HIV amongst drug users in Qingdao in 2016.

Questions	Number of correct answers (%)
Can a person looking healthy in appearance be an HIV carrier?	337(56.2%)
Can HIV be transmitted by bloodtransfusion?	523(87.2%)
Can HIV be transmitted by sharing needles with an HIV carrier or AIDS case?	523(87.2%)
Can the risk of HIV transmission be reduced by the proper use of condoms?	477(79.5%)
Can the risk of HIV transmission be reduced by keeping one sexual partner?	478(79.7%)
Can HIV be transmitted to the foetus by an infected pregnant mother?	474(79.0%)
Can HIV be transmitted by eating together with an HIV carrier or AIDS case?	423(70.5%)
Can HIV be transmitted by mosquito bite?	189(31.5%)

### Drug use behaviour amongst heterosexual drug users

The average onset age of drug use amongst the participants was 28.2 ± 9.3 (12–55) years. According to the types of drugs used, 554 (92.3%) participants used MA, 61 (10.2%) heroin, 32 (5.3%) ketamine, 18 (3.0%) marijuana, 11 (1.8%) dolantin, 10 (1.7%) ecstasy and 9(1.5%) other drugs in the past year. Furthermore, 569 (94.8%) used only one kind of drug, 31 (5.2%) used more than one kind of drug and 14 (2.3%) shared needles with others in the past year. As to the routes of drug use, 546 (91.0%) used by non-injection routes only and 54 (9.0%) by injection or both.

### High risk sexual behaviour amongst heterosexual drug users

Amongst the 600 participants, 322 (53.7%) had sex with casual partners, 335 (55.8%) paid for sex and 92 (15.3%) sold sex for money in the past year.

### Health seeking behaviour amongst heterosexual drug users

Amongst the 600 participants, 129 (21.5%) had STI symptoms, including urodynia or burning pain during urination, abnormal urethral or vaginal discharge, genital erosion or ulcer or neoplasm. To cope with the symptoms, 53 (41.1%) participants visited general hospitals, 25 (19.4%) performed self-medication, 24 (18.6%) visited special hospitals for skin disease, 21 (16.3%) visited private clinics and 12 (9.3%) disregarded the symptoms. In addition, 43 (7.2%) received treatment in addiction treatment centres in the past year.

Amongst the participants, 49 (8.2%) received free condoms, and 150 (25.0%) received free publicity material from health workers. Furthermore, 520 (86.7%) learned about HIV/AIDS from television, 275 (45.8%) from the press, 176 (29.3%) from broadcasts, 156 (26.0%) from bulletin boards, 148 (24.7%) from books, 140 (23.3%) from free publicity materials, 113 (18.8%) from doctors, 111 (18.5%) from friends, 106 (17.7%) from VCT and 13 (2.2%) from the Internet.

### HIV test uptake and related factors amongst heterosexual drug users

A total of 155 (25.8%) participants underwent HIV testing in the past year, and no positive HIV antibody was found. Amongst these participants, 55 (36.7%) underwent the test at centres for diseases prevention and control(CDCs) and 95 (63.3%) at medical institutions. In univariate analysis, HIV testing was related to certain variables, including marital status (*X*^2^ = 5.896, p = 0.009), education (*X*^2^ = 3.795, p = 0.041), perception of HIV (*X*^2^ = 14.334, p <0.001), onset age of drug use (*X*^2^ = 5.574, p = 0.01), years of drug use (*X*^2^ = 8.167, p = 0.003), types of drugs used (*X*^2^ = 14.354, p <0.001), routes of drug use (*X*^2^ = 10.728, p = 0.001), free condoms received in the past year (*X*^2^ = 27.3, p<0.001) and free publicity materials received in the past year (*X*^2^ = 51.292, p<0.001) (Table 2).

**Table 2:** Univariate analysis of factors related to HIV testing amongst drug users in Qingdao in 2016.

Variable	Total	HIV testing		*X*^2^	p
	*N* = 600	Yes (*n*, %)	No (*n*, %)		
1.Demographic information					
Age					
<30	254	62(24.4%)	192(75.6%)	0.466	0.510
≥30	346	93(26.9%)	253(73.1%)		
Gender					
Male	488	120(24.6%)	368(75.4%)	2.109	0.152
Female	112	35(31.3%)	77(68.8%)		
Marital status					
Married	435	124(28.5%)	311(71.5%)	5.896	0.009
Single	165	31(18.8%)	134(81.2%)		
Residency					
Permanent residents	411	109(26.5%)	302(73.5%)	0.322	0.616
Mobile population	189	46(24.3%)	143(75.7%)		
Ethnicity					
Han	475	152(26.5%)	422(73.5%)	2.899	0.063
Others	26	3(11.5%)	23(88.5%)		
Education					
College or above	51	19(37.3%)	32(62.7%)	3.795	0.041
High school or below	549	136(24.8%)	413(75.2%)		
Occupation					
Unemployed	260	65(25.0%)	195(75.0%)	0.166	0.683
Employed	340	90(26.5%)	250(73.5%)		
2. Perception of HIV					
≥6	398	122(30.7%)	276(69.3%)	14.334	<0.001
<6	202	33(16.3%)	169(83.7%)		
3. Drug use behaviour					
Onset age of drug use					
<40	506	140(27.7%)	366(72.3%)	5.574	0.010
≥40	94	15(16.0%)	79(84.0%)		
Years of drug use					
≥5	281	88(31.3%)	193(68.7%)	8.167	0.003
<5	319	67(21.1%)	251(78.9%)		
Types of drugs used					
Multiple	31	17(54.8%)	14(45.2%)	14.354	<0.001
Single	569	138(24.3%)	431(75.7%)		
Routes of drug use					
Injection	54	24(44.4%)	30(55.6%)	10.728	0.001
Non-injection only	546	131(24.0%)	415(76.0%)		
4. Sexual behaviour					
Having sex with casual partners in the past year					
Yes	322	86(26.7%)	236(73.3%)	0.278	0.333
No	278	69(24.8%)	209(75.2%)		
Buying sex in the past year					
Yes	335	82(24.5%)	253(75.5%)	0.728	0.400
No	265	73(27.5%)	192(72.5%)		
Selling sex in the past year					
Yes	92	31(33.7%)	61(66.3%)	3.506	0.070
No	508	124(24.4%)	384(75.6%)		
5. Health seeking behaviour					
Having symptoms of STI in the past year					
Yes	129	41(31.8%)	88(68.2%)	3.036	0.089
No	471	114(24.2%)	357(75.8%)		
Receiving free condoms in the past year					
Yes	49	28(57.1%)	21(42.9%)	27.3	<0.001
No	551	127(23.0%)	424(77.0%)		
Receiving free publicity materials in the past year					
Yes	150	72(48.0%)	78(52.0%)	51.292	<0.001
No	450	83(18.4%)	367(81.6%)		

Multivariate analysis results revealed that HIV test uptake is positively associated with marital status (adjusted OR(AOR) = 1.923, 95% CI = 1.189–3.109), perception of HIV (AOR = 1.706, 95% CI = 1.074–2.711), types of drugs used (AOR = 2.649, 95% CI = 1.155–6.077), routes of drug use (AOR = 2.121, 95% CI = 1.103–4.078), free condoms received in the past year (AOR = 2.307, 95% CI = 1.129–4.715) and free publicity materials received in the past year (AOR = 2.828, 95% CI = 1.757–4.552). Drug users who were single, had less knowledge of HIV, used only one kind of drug, used the drug via a non-injection route, did not receive free condoms and free publicity materials from health workers in the past year were less likely to undergo HIV testing (Table 3).

**Table 3:** Multivariate analysis factors related to HIV testing amongst drug users in Qingdao in 2016.

Variable	OR (95%CI)	p
Marital status (0 = Married,1 = Single)	1.923 (1.189–3.109)	0.008
Education(0 = College or above,1 = High school or below)	1.159 (0.593–2.265)	0.667
Perception of HIV (0 = ‘≥6’,1 = ‘<6’)	1.706 (1.074–2.711)	0.024
Onset age of drug use (0 = ‘<40’,1 = ‘≥40’)	1.473 (0.772–2.809)	0.240
Years of drug use (0 = ‘≥5’,1 = ‘<5’)	1.276 (0.824–1.975)	0.274
Drugs ever used (0 = Multiple,1 = Single)	2.649 (1.155–6.077)	0.021
Types of drug use (0 = Injection,1 = Non-injection)	2.121 (1.103–4.078)	0.024
Receiving free condoms from health workers in the past year (0 = Yes,1 = No)	2.307 (1.129–4.715)	0.022
Receiving free publicity materials from health workers in the past year (0 = Yes,1 = No)	2.828 (1.757–4.552)	<0.001

### Results of HIV and syphilis screening amongst heterosexual drug users

This study provided HIV and syphilis screening for the participants. Nearly all the participants (594, 99.0%) underwent the test. Meanwhile, two participants (0.3%) were HIV antibody positive and 88 (14.8%) were both RPR and TPPA positive for the first time. The two cases that were HIV antibody positive were confirmed with Western blot at the municipal CDC.

## Discussion

The current study aimed to investigate the utilisation of HIV testing and related factors that influence heterosexual drug users, particularly non-injection drug users, in China to undergo HIV testing. The results showed that the rate of HIV test uptake amongst heterosexual drug users was 25.8%. Zhao et al. reported that among MSM, 69% never had an HIV test in the past year [23]. Moreover, 70% of substance users who previously tested as negative in an HIV test took another HIV test in the past year [24]. In the present study, the rate of heterosexual drug users taking an HIV test was lower than that amongst MSM in China and heterosexual drug users in other countries.

Several factors related to HIV testing amongst drug users were determined by multivariate analysis. Injection drug users who were married were less likely to undergo HIV testing [25]. The present study determined that heterosexual drug users, particularly non-injection drug users, and who were single were less likely to undergo HIV testing. Lack of information is one of the factors that account for the low utilisation of HIV testing [21]. Zhou et al. reported that 75.2% of VCT clients from a high-risk area in China were motivated to undergo HIV testing after they acquired knowledge of HIV [26].The results of the present study were consistent with those of previous studies. We usually noticed the separation of HIV/AIDS knowledge and behaviour and failure of intervention amongst high risk groups, especially amongst MSM. The current study found that heterosexual drug users who had less knowledge on HIV and did not receive free condoms and publicity materials from health workers in the past year were less likely to undergo HIV testing, indicating that health education and promotion are effective measures to improve HIV test uptake amongst heterosexual drug users. Moreover, the types and routes of drug use were associated with HIV test uptake. The current study also observed that heterosexual drug users who used only one kind of drug and via a non-injection route in the past year were less likely to undergo HIV testing. Most of the participants of the study used MA through the non-injection route only, indicating that MA users were less likely to undergo HIV testing than injection drug users. Saw et al. reported that lesser non-injection drug users (46%) tested for HIV compared with injection drug users (77%) [25]. The high OR of HIV testing amongst injection drug users may be the effect of the scale-up intervention for IDUs across China since 2004.Thus, considerable effort is needed to improve HIV test uptake amongst new types of drug users.

A relatively low prevalence of HIV infection was observed amongst heterosexual drug users, in spite of the high prevalence of syphilis, indicating a favourable opportunity to conduct specific intervention amongst this population. We suggest that a comprehensive intervention programme should be conducted in this population, including social mobilisation, health promotion, STI screening and treatment, and non-governmental organisations involvement.

Several limitations of this study should be considered when interpreting the findings. Firstly, the study population was recruited from a detention centre and community. Most of the participants were non-injection drug users and were not randomly recruited. Meanwhile, homosexual drug users who had higher risk for HIV were not covered by this study due to limited sample available. Therefore, the sample may not be representative of the general population of drug users in China. Secondly, participants from the detention centre might have been afraid that their information was exposed, which may have lead to reporting bias. Lastly, the cross-sectional study might not provide causal inference; the related factors that we found were not necessarily to cause HIV testing uptake behaviour, and many barriers that may prevent heterosexual drug users from undergoing HIV testing might not have been included in this study.

## Conclusions

The rate of HIV test uptake amongst heterosexual drug users in China remains low. Thus, more specific interventions are urgently needed to improve the utilization of HIV testing amongst drug users, particularly amongst non-injection drug users, in China.

## Supporting information

S1 Text (DOCX)

S2 Text (DOCX)

## Abbreviations

AOR: adjusted odds ratio
CI: confidence interval
MA: methamphetamine
MSM: men who have sex with men
RPR: rapid plasma regain test
STI: sexually transmitted infections
TPPA: *treponema pallidum* particle agglutination test
VCT: voluntary counseling and testing
