# Supplementary material for: HIV test uptake and related factors amongst heterosexual drug users in Shandong province, China
Source: PLoS One. 2018 Oct 18;13(10):e0204489. doi: 10.1371/journal.pone.0204489 (PMC6193625; doi:10.1371/journal.pone.0204489)
Supplement: S1 Text — (DOCX) [file pone.0204489.s001.docx]

**Informed consent form**

*Ms/Mr. XXX:*

*I am Dr. X, working for the XX.*

*I am doing a study on health problem of drug users. I am going to give you information and invite you to be part of this research. HIV and AIDS have been spreading among drug users. We want to find ways to stop this from happening. We believe that you can help us by telling us what you know about drug abuse and about your concepts and behaviors related to HIV and AIDS. This study will involve your participation in an interview that will take about half an hour. Your participation in this research is entirely voluntary. It is your choice whether to participate or not. Your response or rejection has none relation to your crimes and punishment.*

*We also want to know your status of HIV and syphilis infection, what influenced you to take HIV testing and how serious STD and HIV/AIDS are spreading in this population, so after the interview you are welcome to take an examination. All the services provided are free and you welcome anytime. We are asking you to share with us some very personal and confidential information, and you may feel uncomfortable talking about some of the topics. During the interview, if you do not wish to answer any of the questions during the interview, you may say so and I will move on to the next question. There will be no direct benefit to you, but your participation is likely to help us find out more about how to improve HIV screening in the population. The information recorded is confidential, and no one else except our work staff will access to the information documented during your interview. If you have any questions, you can ask us now or later. If you wish to ask questions or have an examination later, you may contact Dr. Jiang Zhenxia, Qingdao Municipal Center for Disease Control and Prevention, her telephone number is 18853280531.*
