# Supplementary material for: HIV test uptake and related factors amongst heterosexual drug users in Shandong province, China
Source: PLoS One. 2018 Oct 18;13(10):e0204489. doi: 10.1371/journal.pone.0204489 (PMC6193625; doi:10.1371/journal.pone.0204489)
Supplement: S2 Text — (DOCX) [file pone.0204489.s002.docx]

**Questionnaire for drug users**

*Ms/Mr. XXX:*

*I am Dr. X, working for the XX.*

*I am doing a study on health problem of drug users. I am going to give you information and invite you to be part of this research. HIV and AIDS have been spreading among drug users. We want to find ways to stop this from happening. We believe that you can help us by telling us what you know about drug abuse and about your concepts and behaviors related to HIV and AIDS. This study will involve your participation in an interview that will take about half an hour. Your participation in this research is entirely voluntary. It is your choice whether to participate or not. Your response or rejection has none relation to your crimes and punishment.*

*We also want to know your status of HIV and syphilis infection, what influenced you to take HIV testing and how serious STD and HIV/AIDS are spreading in this population, so after the interview you are welcome to take an examination. All the services provided are free and you welcome anytime. We are asking you to share with us some very personal and confidential information, and you may feel uncomfortable talking about some of the topics. During the interview, if you do not wish to answer any of the questions during the interview, you may say so and I will move on to the next question. There will be no direct benefit to you, but your participation is likely to help us find out more about how to improve HIV screening in the population. The information recorded is confidential, and no one else except our work staff will access to the information documented during your interview. If you have any questions, you can ask us now or later. If you wish to ask questions or have an examination later, you may contact Dr. Jiang Zhenxia, Qingdao Municipal Center for Disease Control and Prevention, her telephone number is 18853280531. If you agree, please give your signature.*

*Thank you for your cooperation.*

*Signature:*

-------------------------------------------------------------------------------------------------------

**A--registration**

Registration Number □□□

A01 Date of interview year□□□□month□□date□□

A02 Sites of recruitment ① community ②municipal detention centre □

-------------------------------------------------------------------------------------------------------

**B--General information**

B01 Sex ①Male ②Female □

B02 Birthday year □□□□month□□

B03 Marital status ①Single ②Married ③Divorced ④widowed □

B04 Ethnicity ①Han ②Others □

B05 Education □

①Elementary school ②Junior school ③High/technical school

④College or above ⑤illiterate

B06 Registered residence □

①Permanent residents ② other city in Shandong ③ other province

B07 Employment ①Employed ②Unemployed □

-------------------------------------------------------------------------------------------------------

**C-- Perception of HIV/AIDS**

C01 Can a person looking healthy in appearance be an HIV carrier? □

①Yes ②No ③I don’t know

C02 Can HIV be transmitted by [bloodtransfusion](javascript:void(0);)? □

①Yes ②No ③I don’t know

C03 Can HIV be transmitted by sharing needles with an HIV carrier? □

①Yes ②No ③I don’t know

C04 Can the risk of HIV transmission be reduced by the proper use of condoms? □

①Yes ②No ③I don’t know

C05 Can the risk of HIV transmission be reduced by keeping one sexual partner?□

①Yes ②No ③I don’t know

C06 Can HIV be transmitted to the foetus by an infected pregnant mother? □

①Yes ②No ③I don’t know

C07 Can HIV be transmitted by eating together with an HIV carrier? □

①Yes ②No ③I don’t know

C08 Can HIV be transmitted by mosquito bite? □

①Yes ②No ③I don’t know

-------------------------------------------------------------------------------------------------------

**D--Behaviors of drug use**

D01 How old was you when you first used drug? □□

D02 How did you use drugs in the past year? □

①Injection ②Non-injection ③Both

D03 What kinds of drugs have you ever used in the past year? □□□

### ①MA ②Heroin ③ketamine ④Marijuana ⑤Dolantin ⑥Ecstasy ⑦Others

### (Multiple choice)

D04 Did you shared needles with other in the past year? □

①Yes ②No

-------------------------------------------------------------------------------------------------------**E-- High risk sexual behaviors**

E01 Have you ever had sex with casual partners in the past year? □

①Yes ②No

If yes

E02 Have you paid money to casual partners for sex in the past year? □

①Yes ②No

E03 Have you ever had sex with casual partners for money in the past year? □

①Yes ②No

-------------------------------------------------------------------------------------------------------

**F--Health seeking behaviour**

F01 Did you have the follow symptoms of STI in the past year? □□□

①Urodynia ②Burning pain during urination ③Abnormal urethral or vaginal discharge ④Genital erosion or ulcer ⑤Neoplasm ⑥None (Multiple choice)

If yes

F02 How did you usually deal with it? □

①visited [general](javascript:;) hospitals ②visited special hospitals for skin disease

③visit private clinics ④self-medication ⑤let it alone

F03 Did you received treatment in addiction treatment centres in the past year? □

①Yes ②No

F04 Did you received the following preventive health services in the past year? □

①free condom ②publicity material ③None

F05 What is the source for you to get HIV/AIDS information? □□□

①Television ②The press ③Broadcasts ④Bulletin boards ⑤Books

⑥Free publicity materials ⑦Doctors ⑧Friends ⑨VCT ⑩The Internet

(Multiple choice)

F06 Did you received HIV antibody testing in the past year? □

①Yes ②No

If yes

F07 Where was the test taken? □

①Centres for diseases prevention and control ②Medical institutions

-------------------------------------------------------------------------------------------------------

**吸毒人群健康调查问卷**

XXX 先生/女士:

我是来自XX 的 X医生。

我们正做一项关于吸毒人群的健康调查。我将介绍一下这个项目，希望您能参加。艾滋病已在吸毒人群流行，我们想找到减少流行的方法。我们希望你告诉您一些你与艾滋病和吸毒有关的认识和行为。这个访谈需要大约半个小时时间。这个调查是完全自愿的，您可以选择是否参加，您的选择对您个人没有任何影响。

我们也想知道您是否感染艾滋病和梅毒，什么因素影响您参加艾滋病和梅毒检测。访谈结束后我们会给您提供免费检测。我们需要您提供个人隐私方面的信息，可能使您感到尴尬。如果访谈过程中，某些问题您不想回答，我们可以问下一个问题。这次访谈对您个人没有直接益处，但您提供的信息可帮助我们促进吸毒人群艾滋病的筛查。您提供的信息都是保密的，除项目工作人员外，别人不会知道。如果您有什么疑问，可现在或过后咨询我们。您可以联系市疾控中心姜珍霞医师咨询或寻求检测服务，她的电话号码是18853280531. 如果同意，请签名。

谢谢您的合作！

签名：

-------------------------------------------------------------------------------------------------------

**A—登记**

登记号码 □□□

A01 访谈日期 □□□□年□□月□□日

A02 地点 ① 社区 ②市看守所 □

-------------------------------------------------------------------------------------------------------

**B—基本信息**

B01 性别 ①男性 ②女性 □

B02 出生日期 □□□□年□□月

B03 婚姻 ①未婚 ②已婚 ③离婚 ④丧偶 □

B04 民族 ①汉族 ②其它 □

B05 教育水平 □

①小学 ②初中 ③高中/技校

④大学及以上 ⑤文盲

B06 户籍 □

①本地 ②本省 ③外省

B07 就业状况 ①就业 ②无业 □

-------------------------------------------------------------------------------------------------------

**C—对艾滋病的认知**

C01一个看上去健康的人有可能携带艾滋病病毒吗？ □

①能 ②不可能 ③不知道

C02输入带有艾滋病病毒的血液或血液制品会不会感染艾滋病病毒? □

①会 ②不会 ③不知道

C03与艾滋病病毒感染者或病人共用针具会不会感染艾滋病病毒? □

①会 ②不会 ③不知道

C04每次性行为时正确使用安全套能不能降低艾滋病病毒传播的危险？ □

①能 ②不能 ③不知道

C05保持一个未感染艾滋病病毒的性伴能不能降低艾滋病病毒传播的危险？ □

①能 ②不能 ③不知道

C06感染了艾滋病病毒的孕妇有可能将艾滋病病毒传染给她的孩子吗? □

①可能 ②不可能 ③不知道

C07与艾滋病病毒感染者或病人一起吃饭会不会感染艾滋病病毒? □

①会 ②不会 ③不知道

C08蚊虫叮咬会不会传播艾滋病病毒？ □

①会 ②不会 ③不知道

-------------------------------------------------------------------------------------------------------

**D—吸毒行为**

D01 你的首次吸毒年龄是多少? □□

D02 近一年你的吸毒方式是那种? □

①注射 ②非注射 ③都有

D03 过去一年你主要使用那种毒品? (多选) □□□

①冰毒 ②海洛因 ③K粉 ④大麻 ⑤杜冷丁 ⑥摇头丸 ⑦其它

D04 过去一年你与别人共用过针具吗? □

①是 ②否

-------------------------------------------------------------------------------------------------------**E—高危性行为**

E01 过去一年你与偶遇性伴发生过性关系吗? □

①是 ②否

如果是

E02 过去一年你有过付钱与偶遇性伴发生性关系吗? □

①是 ②否

E03 过去一年你有过收钱与偶遇性伴发生性关系吗？ □

①是 ②否

-------------------------------------------------------------------------------------------------------

**F—求医（健康）行为**

F01 过去一年你有过下列性病有关症状吗? (多选) □□□

①尿痛 ②排尿灼热感 ③尿道/阴道分泌物异常

④生殖器糜烂或溃疡 ⑤赘生物 ⑥无

如果有

F02 你是如何处理的? □

①综合医院就诊 ②皮肤病专科医院就诊

③私立诊所就诊 ④自购药治疗 ⑤未处理

F03 过去一年你在戒毒中心治疗过吗? □

①是 ②否

F04 过去一年你接受过以下预防性卫生服务吗? □

①免费安全套 ②宣传资料 ③无

F05 你通过哪些途径获得艾滋病防治知识? (多选) □□□

①电视 ②报刊 ③广播 ④宣传栏 ⑤书籍 ⑥免费宣传资料

⑦医生 ⑧朋友 ⑨自愿咨询检测VCT ⑩互联网

F06 近一年你做过艾滋病抗体检测吗? □

①是 ②否

如果是

F07 在哪里做的? □

①疾控中心 ②医疗机构

-------------------------------------------------------------------------------------------------------
